# Effect of Different Environment Enrichments on Behaviour and Social Interactions in Growing Pigs

**DOI:** 10.3390/ani9030101

**Published:** 2019-03-19

**Authors:** Lorella Giuliotti, Maria Novella Benvenuti, Alessandro Giannarelli, Chiara Mariti, Angelo Gazzano

**Affiliations:** Department of Veterinary Science, University of Pisa, Viale delle Piagge, 2, 56124 Pisa, Italy; novella.benvenuti@unipi.it (M.N.B.); a.giannarelli@studenti.unipi.it (A.G.); chiara.mariti@unipi.it (C.M.); angelo.gazzano@unipi.it (A.G.)

**Keywords:** pig, environmental enrichment, behaviour, social interactions

## Abstract

**Simple Summary:**

Pigs reared under intensive conditions are subjected to environmental stresses such as being unable to express some natural behaviours, like socialisation, exploring and rooting. For this reason, EU legislation requires farmers to employ suitable environmental enrichments in the pens. This study aimed at evaluating how different environmental enrichment tools (wooden logs either hanging or laying and hanging metal chains in pens) affected the behaviour of growing pigs. The results show a reduction in the incidence of aggressive/damaging interactions between animals in the pen where hanging wooden logs were placed. No significant effect on non-aggressive behaviours was noted in any of the investigated conditions.

**Abstract:**

(1) Background: Pigs are active animals that require a suitable environment to be able to express their exploratory behaviour. The aim of the present study was to compare the effects of different environmental enrichments on the behaviour, social interactions, salivary cortisol concentration and body weight of pigs during the growing phase. (2) Methods: The investigation involved 75 pigs divided into three groups. The environmental enrichments were arranged as follows: Hanging metal chains for the control group; hanging metal chains and hanging logs for the second group; hanging metal chains and logs laying on the floor for the third group. Each group was video recorded twice a week for six weeks. The scan sampling technique was used. Salivary cortisol and live body weight were also recorded regularly. Parametric (ANOVA) and non-parametric statistics were used to analyse the data. (3) Results: Hanging logs were found to be more effective than logs laying on the floor at reducing aggression within the group tested, resulting in a more comfortable environment. Salivary cortisol concentration and growth did not show significant differences between the three groups. (4) Conclusions: The use of hanging logs affected some interactive patterns that resulted in decreasing the aggressive episodes of pigs, thereby providing a more comfortable environment.

## 1. Introduction

Pigs have an innate propensity for socialisation, exploration, rooting and chewing behaviours. When individuals are unable to express these behaviours, e.g., in poorly enriched environments, abnormal activities may surface [1].

The main housing solutions adopted in the post-weaning and fattening swine sectors are generally not geared up to satisfy the need for expression of these natural behaviours of the species. A monotonous environment can cause stress and induce stereotypic behaviours and apathy, which can then give rise to extremely dangerous phenomena, such as biting the tails and ears of pen-mates [2]. For this reason, environmental enrichments can improve pig welfare by reducing the incidence and severity of behavioural alterations.

EU legislation [3] requires commercial producers to allow pigs to have permanent access to a sufficient quantity of manipulating material, such as straw, hay, wood, sawdust, mushroom compost or peat. However, specific advice on how to provide this enrichment is lacking. In fact, pig farmers must provide such suitable enrichment materials in a way that is compatible with the animal waste handling system, and takes into consideration the cost implications and the impact on animal health [4,5]. Dangling metal chains are commonly used in intensive European pig farms as a form of enrichment [6]. However, this enrichment is not recommended for long-term use, because it quickly loses its novelty, and pigs lose interest in it [7].

What might be the most effective enrichment to improve pig welfare is still debated. This study aims to compare the effects of different environmental enrichments on the behaviour, social interactions, salivary cortisol concentration and body weight of pigs during the growing phase.

## 2. Materials and Methods

All procedures and treatments were in compliance with the EU Directive 2001/88/EC [8] and EU Directive 2001/93/EC [3] regarding minimum standards for the protection of pigs. Although chains are not entirely considered an adequate enrichment according to EU legislation, they were included in this paper as a control in accordance with Italian legislation [4], which allows the use of other materials when a risk to the functionality of the system exists.

The study was conducted in a farrow-to-finish herd located in the district of Pisa, Italy, from October to December 2016. Seventy-five Goland hybrid grower pigs of both sexes (females and castrated males) were enrolled in the experiment. All subjects were housed in the same building equipped with concrete-slatted-floors pens (2.4 × 6.1 m) and an automatically controlled natural ventilation system. The animals were checked twice a day, and artificial lighting was provided from sunset to 8 pm. The pigs had free access to water from two nipple drinkers per pen, and wet meals were offered every 3 h from 7 am to 7 pm (5 meals/day in total).

A set of dangling metal chains, usually adopted in the farm as environmental enrichment, were present in each pen. The 75 subjects were divided into three groups of 25 each. These groups were homogeneous in body weight (34.9 ± 2.57 kg), sex (13 females and 12 castrated males) and age (11 weeks). The animals were randomly assigned to the three groups:

- Control (C): only dangling metal chains were offered to the animals.
- Hanging logs (HL): three small logs of wood (30 cm) were hooked to the metal chains and hung at 60 cm from the ground.
- Laying logs (LL): three small logs of woods (30 cm) were placed on the floor. Subjects in this group were thus able to interact with both the logs and the dangling metal chains.

The logs were made of poplar, which was selected thanks to its being suitable for chewing and manipulation, harmless, readily available, economical and easy to fit into the farming routine. The wooden enrichment was introduced at the beginning of the trial; as they deteriorated, the logs in LL were replaced almost every two days while the logs in HL were never replaced.

The trial began after a one-week adaptation period. Videos were recorded twice a week for a total period of six weeks by means of Go-Pro Hero cameras placed in front of each of the three pens to gain a complete view of the subjects. On each day of observation, two 90-min recording sessions took place, one in the morning (around 09:00) and one in the afternoon (around 15:00), far enough from the feeding time to ensure a good level of activity and to minimise confounding due to eating behaviour.

Observations of non-social behaviours and social interactions were conducted blind by the same trained observer throughout the experiment. Behavioural observations were carried out according to the scan sampling technique [9], i.e., 30-s observations every 5 min, and the number of subjects that were performing the specific actions outlined in Table 1 was reported.

The number and type of social interactions were recorded by applying the behaviour sampling method: the same videos were fast-forwarded and paused at the moment in which two or more pigs came into contact with one another. The social interactions (Table 2) were assembled on the basis of the ethogram outlined by Jensen [10]. Moreover, in order to facilitate data analysis, the social interactions were categorised as either “aggressive/damaging” or “non-aggressive” [11,12].

To appraise the evolution of non-social behaviours and social interactions during the trial, the experimental period was divided into three phases of 2 weeks each: Initial (1st and 2nd week), mid-term (3th and 4th week) and final (5th and 6th week).

At the beginning of the trial and every two weeks, salivary cortisol samples were gathered in each pen for a total of four samplings. The samples were collected using large cotton swabs deposited in the pen. These swabs were randomly chewed by the pigs for 30 s and were withdrawn immediately afterwards. The samples were preserved at 4 °C in tubes until they arrived at the Etovet laboratory of the Department of Veterinary Science of the University of Pisa to be stored at −20 °C until the analysis. Every sample was assayed for salivary cortisol using an enzyme immunoassay kit (Diametra^®^ Cortisol Saliva, Spello, Perugia, I).

The body weight of all pigs was measured at the beginning and at the end of the trial. During the experiment, some subjects (three pigs from the HL group, three from the LL group and five from the C group) were removed for illness and the data were promptly updated. Results for non-social behaviours and social interactions were expressed as the percentage of the total number of pigs who performed an activity.

Statistical analyses were performed by the SAS-JMP software [13], as follows:

- ANOVA for the behavioural data; the model included group, period and observation time (morning or afternoon) as variables;
- Wilcoxon nonparametric test for social interactions;
- ANOVA for final body weight and salivary cortisol, using the group as variability factor and initial body weight as covariate only in the former.

A *p*-value ≤ 0.05 was considered significant.

## 3. Results and Discussion

### 3.1. Behavioural Data

Regarding the behavioural differences between the groups (Table 3), statistical analysis uncovered significant differences for “social activity” and “enrichment examination” (*p* ≤ 0.01), as well as on “pen exploration” and “log examination” (*p* ≤ 0.05).

The “pen exploration” trend in C and HL was in accordance with data reported by Beattie et al. [14], whose studies indicated that environmental enrichment increased the time spent in exploratory behaviour. The same authors also reported a positive effect of environmental enrichment on active behaviours, but in our study this effect did not reach statistical significance.

“Social activity” was significantly lower in LL; in fact, the presence of material that could be manipulated seemed to distract the animals from social interactions (both aggressive/damaging and non-aggressive), and this effect was particularly pronounced in the LL group, where the animals could effectively avail themselves of both logs and chains. This result was in accordance with the study of Beattie et al. [14], who affirmed that persistent interactions among pigs represent a redirected impulse of environment manipulation.

“Enrichment examination” was higher in the HL and LL than in the C group, in agreement with Telkänranta et al. [15], who found that pigs prefer to manipulate wooden logs than chains. “Log examination” tended to be higher in HL than in LL. The lower interest of the animals in the LL pen in the wooden logs can be explained by the fact that the logs might be soiled with faeces, becoming unappealing to the animals, as hypothesised by Battini et al. [16].

The most commonly observed behaviours were “laying inactive” followed by “pen exploration” and “eating,” in agreement with Cornale et al. [17], who observed that pigs spent most of their time resting on the floor (>50%), exploring the pen and standing at the through.

### 3.2. Social Interactions

The statistical analysis showed significant differences in social interactions (Table 4) between the groups for the variables “head-to-head knock,” “biting ear or tail,” “belly nosing,” and “aggressive interactions.”

“Aggressive/damaging interactions” were higher in the C group than in HL. This difference was mainly caused by the values of the parameters “head-to-head knock” and “ear and tail biting“. Similar results were observed in a trial conducted by Cornale et al. [17], in which pigs reared in unenriched pens showed higher rates of tail biting and aggression compared to pigs reared in pen equipped with hanging wooden logs. Other studies detected a reduction in the incidence of aggression among pigs reared in an enriched environment [18,19]. However, there are also studies that show higher levels of aggressive activities in pigs reared in enriched environments [20].

“Belly nosing” was the greatest in HL, lower in C and lower still in LL. The possible cause of belly nosing has not yet been clarified. It has been suggested that one of the principal causes can be the early age of weaning [21]. Therefore, it has been hypothesised that the social environment can also have a profound effect on the incidence of belly nosing [22].

### 3.3. Behavioural Observation and Social Interactions by Period

Variations in behaviour along the trial are summarised in Table 5. Significant differences were recorded for “log examination” (*p* ≤ 0.01) as well as for the variables “drinking” and “pen exploration” (*p* ≤ 0.05).

Petersen et al. [23] reported that eating behaviour increased substantially across time concurrently with the age of the animal, while in the present study “eating” remained steady during the trial. “Log examination” followed a decreasing trend in the final period of the trial, possibly due to the loss of interest in the enrichment over time, as found by Van de Weerd et al. [24].

Table 6 reports the time variation of social interactions over time.

Belly nosing showed a decreasing trend during the study. This was in accordance with the findings of Torrey and Widowsk [21], who defined this activity as a transient pattern, representing a redirected suckling behaviour in confined pigs.

“Parallel pressing” was significantly variable during the trial period, while “anogenital nosing” showed a decreasing trend. The later interaction represents a mechanism of mutual recognition [10]. Thus, it is hypothesised that the decrease reflects greater acquaintance and a relatively more stable hierarchy among the pigs in the final period.

### 3.4. Observation during the Day

Table 7 reporting the behavioural changes across the day reveals that all the observed parameters that showed statistical differences.

Every behaviour significantly differed across the time of the day, showing greater activity in the afternoon. Consequently, parameters such as “standing inactive” and “laying inactive”, representing inactivity, were prevalent in the morning. This general pattern was also recorded by Fraser et al. [25], who observed that pigs showed greater levels of activity in the afternoon rather than in the morning.

Significant differences in social interaction were observed across time of day as reported in Table 8. 

Every interaction was more frequent in the afternoon than in the morning in all groups; these outcomes are in accordance with the findings reported by Ott et al., [26].

### 3.5. Growing Performance and Cortisol

No significant differences in the final weight among the three groups were found in this study (Table 9). The literature on this issue is controversial: Schaefer et al. [18], Horrell [27] and Beattie et al., [14] found a better growth rate in pigs reared in an enriched environment, whereas Pearce et al. [28] and Blackshaw et al. [29], in accordance with our findings, did not notice any weight gain.

No significant differences in salivary cortisol among the groups and the sampling were found (Table 9 and Table 10).

These results confirm the findings of Cornale et al. [17], who reported that the use of hanging wooden logs did not result in significant differences in faecal corticosteroid levels. De Jong et al. [30] found levels in cortisol concentration near to 8 and 6 ng/mL in the 15-week-old pigs reared in enriched and barren environments, respectively. Comparable values were also observed by Smulders et al. [31] in 14 to 20-week-old piglets.

## 4. Conclusions

This study of swine behaviour in response to enrichment yielded interesting results. In detail, our data suggested that the adoption of hanging wooden logs would allow a reduction in the incidence of aggressive/damaging interactions among the animals. At the same time, there was no significant effect on non-aggressive interactions in any of the investigated conditions.

Regarding the levels of activity throughout the day, as expected, active behaviours and interactions were more frequent in the afternoon than in the morning regardless of the kind of enrichment. The proposed environmental enrichments did not induce significant variation in the growth rate and salivary cortisol.

The use of wooden logs (both hanging and laying) showed a decreasing trend during the trial, possibly due to a decline in interest of the animals towards the items. Overall, the pigs interacted more often with the hanging logs, probably because they were not soiled with faeces like the lying logs.

Although the implementation of hanging wooden logs brought positive results, further investigation is necessary in order to verify whether the interest of the animals can be maintained across the time by modifying the enrichment configuration. Moreover, to highlight the differences due to treatments, the replication of the group should be considered.

Finally, in compliance with the recommendation of the EU legislation and in light of our results, hanging metal chains should be replaced with materials that do not damage the functionality of the waste system.

## Figures and Tables

**Table 1:** Definition of the analysed behaviours.

Behaviour	Definition
Standing inactive	Subjects stay and do not exhibit any behaviours.
Laying inactive	Subjects lay motionless in lateral or sternal recumbency.
Eating	Subjects stand in front of the feeder and put their head in contact with the feeding trough.
Drinking	Subjects stand and either touch or play with the nipple drinkers.
Social activity	Subjects chase, shove or scratch pen-mates with their snout, bite ears, feet or tails of pen-mates and perform other aggressive behaviours.
Pen exploration	Subjects move around the pen rooting about the floor.
Enrichments examination	Subjects smell, chew, suck or play with the enrichments.
Log examination	Subjects smell, chew, suck or play with the logs that are either hanging from the chains or laying on the floor

**Table 2:** Description of the observed social interactions.

Interaction	Labelling	Definition of the Interaction
Parallel pressing	Aggressive/damaging	The pigs stand side by side and shove one another until one attempts to bite the other’s head, neck or flank.
Inverse pressing	Aggressive/damaging	The pigs stand in front of one another and push with their heads against the other’s neck or flank.
Head-to-head knock	Aggressive/damaging	A pig uses its head or snout to strike another’s head, neck or ears. This action may be followed by a bite.
Head-to-body knock	Aggressive/damaging	A pig uses its head or snout to strike another pig with a quick blow to any body part behind the ears. This action may be followed by a bite.
Ears or tail biting	Aggressive/damaging	A pig chews, sucks or plays with another’s ears or tails.
Belly nosing	Non-aggressive	A pig uses its snout to repeatedly and continuously massage the abdominal or groin area of another pig that is laying down.
Nose-to-nose	Non-aggressive	A pig places its snout near the head, ears or nose of another pig. A short physical contact may be established.
Nose-to-body	Non-aggressive	A pig places its snout close to the body of another pig, but not in the genital area. A short physical contact may be established.
Anogenital nosing	Non-aggressive	A pig places its snout near the genital area of another. A short physical contact may be established
Withdrawing	Non-aggressive	A pig feels threatened by another, and consequently moves away rapidly while holding its head high and often emitting a shrill cry.

**Table 3:** Behavioural observations (%) by group.

Group	C	HL	LL	Standard Error	*p* Value
Parameter	Mean	Mean	Mean		
Inactive	80.8	76.0	76.7	2.50	0.1688
Active	19.4	24.6	20.6	2.49	0.1447
Standing inactive	0.6	1.0	0.6	0.28	0.3094
Laying inactive	80.2	75.0	76.1	2.57	0.1558
Eating	2.6	2.1	2.0	0.45	0.4363
Drinking	0.4	0.5	0.3	0.90	0.2769
Social activity	1.4 *^A^*	1.2 *^A^*	0.5 *^B^*	0.24	0.0010
Pen exploration	11.9 *^AB^*	14.6 *^A^*	10.2 *^B^*	1.58	0.0413
Enrichments examination	3.0 *^B^*	6.2 *^A^*	7.6 *^A^*	0.94	<0.0001
Log examination	-	6.2	3.3	1.44	0.0524

Different superscript letters in the same row indicate significant differences (B < A). Legend: C (control), HL (hanging logs) and LL (laying logs).

**Table 4:** Observation of social interactions (%) between the three groups.

Group	C		HL		LL		*p* Value
Parameter	Mean	SE	Mean	SE	Mean	SE	
Aggressive/damaging interactions	46.35 *^A^*	6.65	24.10 *^B^*	3.35	32.73 *^AB^*	4.90	0.0265
Non-aggressive interactions	53.59	6.27	59.06	6.02	48.16	4.88	0.3435
Parallel pressing	6.99	1.6	5.86	1.05	5.02	1.11	0.8488
Inverse pressing	4.82	1.07	2.64	0.58	3.51	0.75	0.2260
Head-to-head knock	22.52 *^A^*	2.65	10.76 *^B^*	1.56	15.99 *^B^*	2.05	0.0006
Head-to-body knock	1.29	0.26	1.59	0.39	0.75	0.24	0.1564
Ears or tail biting	10.73 *^A^*	1.59	3.25 *^B^*	0.73	7.46 *^A^*	1.12	0.0002
Belly nosing	5.27 *^B^*	1.29	10.96 *^A^*	2.07	0.76 *^C^*	0.2	<0.0001
Nose-to-nose	20.77	2.84	21.56	2.24	22.80	2.7	0.7802
Nose-to-body	17.03	1.99	13.45	0.82	16.67	0.64	0.3328
Anogenital nosing	7.87	1.10	8.80	0.76	6.27	0.95	0.0620
Withdrawing	2.65	1.47	4.29	0.65	1.66	0.47	0.1232

Different superscript letters on the same row indicate significant differences (B < A). Legend: C (control), HL (hanging logs) and LL (laying logs). SE = standard error.

**Table 5:** Behavioural observations by period (%).

Period	Initial	Mid-Term	Final	Standard Error	*p* Value
Parameter	Mean	Mean	Mean		
Inactive	76.8	75.8	81.0	1.93	0.1299
Active	24.8	24.2	18.8	1.99	0.0700
Standing inactive	0.9	0.7	0.6	0.22	0.7894
Laying inactive	75.9	75.1	80.4	1.99	0.1320
Eating	1.7	2.8	2.1	0.35	0.0944
Drinking	0.3 *^B^*	0.5 *^A^*	0.3 *^B^*	0.07	0.0347
Social activity	1.3	0.9	1.0	0.19	0.2769
Pen exploration	11.8 *^AB^*	14.5 *^A^*	10.4 *^B^*	1.22	0.0562
Enrichment examination	6.9	5.3	4.7	0.72	0.0851
Log examination	9.7 *^A^*	5.5 *^B^*	5.0 *^B^*	0.93	0.0011

Different superscript letters in the same row indicate significant differences (B < A).

**Table 6:** Social interaction by period (%).

Period	Initial		Mid-Term		Final		*p* Value
Parameter	Mean	SE	Mean	SE	Mean	SE	
Aggressive/damaging interactions	32.50	4.19	43.89	7.28	26.82	3.64	0.2681
Non-aggressive interactions	57.67	5.11	54.16	5.53	49.01	5.61	0.3643
Parallel pressing	5.58 *^AB^*	0.10	8.74 *^A^*	1.67	3.55 *^B^*	0.76	0.0592
Inverse pressing	2.33	0.46	5.49	1.00	3.15	1.70	0.1321
Head-to-head knock	16.79	2.05	19.26	3.03	13.23	1.59	0.4315
Head-to-body knock	1.13	0.27	1.69	0.40	0.82	0.20	0.4376
Ears or tail biting	6.67	1.07	8.71	1.67	6.07	1.20	0.5496
Belly nosing	9.92 *^A^*	2.33	3.66 *^B^*	1.03	3.42 *^B^*	0.79	0.0049
Nose-to-nose	19.25	1.64	24.90	2.67	20.99	3.15	0.3189
Nose-to-body	15.83	0.85	15.03	1.57	16.30	2.11	0.4766
Anogenitals nosing	10.17 *^A^*	0.76	6.89 *^B^*	0.55	5.88 *^B^*	0.70	0.0013
Withdrawing	2.50	0.61	3.68	0.86	2.42	0.72	0.3206

Different superscript letters on the same row indicate significant differences (B < A).

**Table 7:** Behavioural observation by time of day (%).

Time of Day Parameter	Morning	Afternoon	Standard Error	*p* Value
	Mean	Mean		
Inactive	90.2	69.9	2.23	<0.0001
Active	13.0	29.9	1.70	<0.0001
Standing inactive	4.9	1.0	0.25	0.0445
Laying inactive	85.3	68.9	2.30	<0.0001
Eating	1.3	3.1	0.40	<0.0001
Drinking	0.2	0.5	0.08	<0.0001
Social activity	0.6	1.5	0.22	0.0002
Pen exploration	7.2	17.3	1.41	<0.0001
Enrichment examination	3.7	7.5	0.84	<0.0001
Log examination	5.0	8.6	1.10	0.0010

**Table 8:** Social interactions at different timed of the day (%).

Time of the Day	Morning		Afternoon		*p* Value
Parameter	Mean	SE	Mean	SE	
Aggressive/damaging interactions	16.13	1.58	51.78	4.54	<0.0001
Non-aggressive interactions	30.55	1.84	76.87	3.30	<0.0001
Parallel pressing	1.50	0.25	10.42	0.99	<0.0001
Inverse pressing	0.10	0.17	6.32	0.71	<0.0001
Head-to-head knock	10.20	0.89	22.65	2.08	<0.0001
Head-to-body knock	0.35	0.11	2.07	0.27	<0.0001
Ears or tail biting	3.98	0.58	10.32	0.13	<0.0001
Belly nosing	2.67	0.55	8.66	1.68	0.0033
Nose-to-nose	11.46	0.78	31.97	1.54	<0.0001
Nose-to-body	10.19	0.66	21.25	1.08	<0.0001
Anogenitals nosing	5.78	0.53	9.71	0.57	<0.0001
Withdrawing	0.45	0.12	5.28	0.62	<0.0001

**Table 9:** Final body weight (kg) and salivary cortisol concentration (ng/mL) of the three groups.

Group	C	HL	LL	Standard Error	*p* Value
Parameter	Mean	Mean	Mean		
Final body weight	56.06	55.65	59.14	4.99	0.8646
Salivary cortisol	4.92	5.97	4.65	0.94	0.6032

Legends: C (control), HL (hanging logs) and LL (laying logs).

**Table 10:** Salivary cortisol concentration (ng/mL) during the trial.

Sampling	1	2	3	4	Standard Error	*p* Value
Parameter	Mean	Mean	Mean	Mean		
Salivary cortisol	6.48	4.86	3.57	5.82	1.09	0.3433
